# Consequences of maternal heat stress at different stages of embryonic and fetal development on dairy cows’ progeny

**DOI:** 10.1093/af/vfab059

**Published:** 2021-12-17

**Authors:** Véronique Ouellet, Alexandra Boucher, Geoffrey E Dahl, Jimena Laporta

**Affiliations:** 1 Department of Animal Sciences, Université Laval, Québec City, QC, Canada; 2 Department of Animal Sciences, University of Florida, Gainesville, FL, USA; 3 Department of Animal and Dairy Sciences, University of Wisconsin–Madison, Madison, WI, USA

**Keywords:** environment, epigenetics, fetal programming, hyperthermia

**Implications**

- Maternal response to late-gestation heat stress alters developmental programming in lactating dairy cattle.
- There is currently limited knowledge on the effects of maternal heat stress occurring during the first months of gestation on postnatal phenotypes in dairy cows.
- Recent evidence indicates that differential DNA methylation arising in utero and intrauterine growth restriction are in part responsible for the long-term altered phenotypes.
- Further research is needed to determine if heat stress exposure during in utero development has direct effects on the germ cells of the developing fetus, leading to phenotype alteration of the granddaughters.

## Introduction

**Figure 1.**
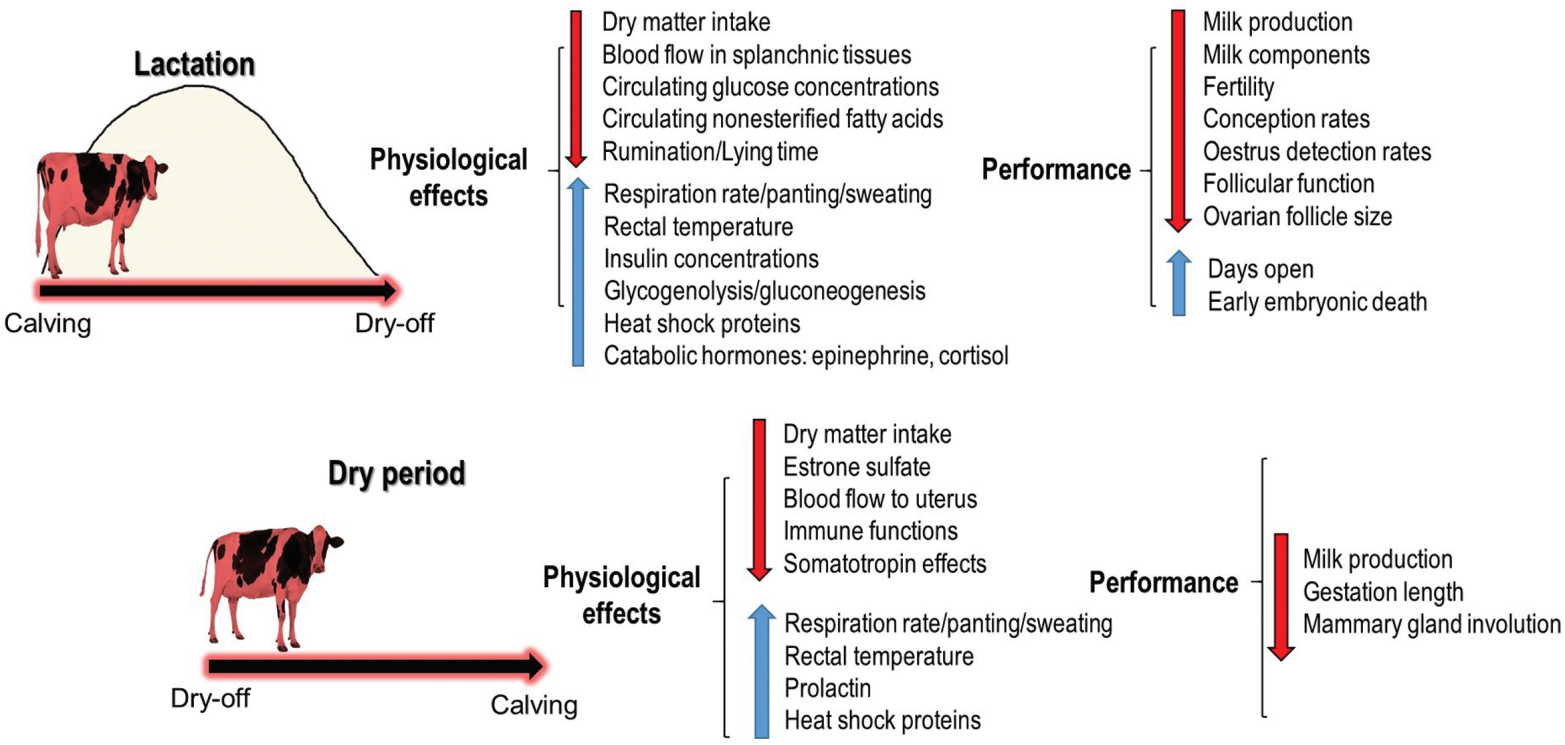
Summary of heat stress-related effects on some physiological parameters and on the performance of dairy cows when heat stress occurs during lactation and the dry period which is defined as the last 6 to 8 wk before calving. The red arrow indicates a decrease, while the blue arrow indicates an increase.

Heat is one of the most important physiological stressors in livestock. Maternal heat stress, defined as an environmentally induced increase in core body temperature above euthermic levels, triggers a series of physiological and behavioral responses (Figure 1) all aimed at decreasing core heat production and/or increasing heat dissipation to the environment (West, 2003). These homeorhetic processes hinder the pregnant dam’s performance (Figure 1) and prenatally expose the offspring to an environment that can trigger lasting epigenetic alterations (Laporta et al., 2020). Prenatal heat stress significantly contributes to global production losses in pigs as it was reported to decrease swine birth weight, increase teratogenicity and core body temperature set point, and alter postnatal body composition (Johnson et al., 2015). Whereas the impacts of prenatal heat stress are well defined in pigs, the effects of a heat insult occurring at critical prenatal stages of development are just becoming apparent in dairy cows.

Dairy cows are gestating during most of their lactation cycle, including the periconceptional period, which includes the period before conception to early gestation, and the dry period, which is commonly defined as a 6- to 8-wk nonlactating phase before calving. While the first months of gestation, coinciding with lactation, are characterized by embryogenesis, placentation, and organ development, the last 2 mo of gestation are concurrent with the dry period and are hallmarked by rapid fetal growth. It is now well established that the maternal intrauterine environment during conception and gestation is determinant for the phenotype of the offspring at adulthood (Bach et al., 2012). In light of this, in utero heat stress (**IUHT**), defined as prenatal exposure of the fetus to maternal body temperatures above euthermic levels, can lead to permanent changes in tissue structure and function, and have detrimental impacts on the subsequent offspring of dairy cows (Brown et al., 2015, 2016; Dado-Senn et al., 2020a). For instance, aberrant mammary gland morphology and the methylation profile of mammary gland DNA have recently been described in late-gestation IUHT heifers, which indicates that epigenetic changes during fetal development may contribute to altered postnatal phenotypes (Skibiel et al., 2018a, 2018b). Along with potential direct epigenetic changes, prenatal heat stress may indirectly impact the developing fetus through intrauterine growth restriction, which can impair the development of the fetus during gestation and have lifelong negative impacts on animal growth and development (Ji et al., 2017). Regardless of the mechanism, maternal heat stress exerts lifelong negative impacts on the resulting offspring that cannot be rescued by postnatal management (Laporta et al., 2020). This review describes the consequences of maternal heat stress at different stages of embryonic and fetal development on the prenatal development, intrauterine and postnatal growth, thermotolerance, metabolism, immune response, and production outcomes of the dairy cows’ progeny. We also discuss underlying physiological mechanisms and identify gaps in current knowledge relevant to epigenetics effects of maternal heat stress.

## Impacts of Maternal Heat Stress on Embryonic Development

Maternal heat stress occurring during the periconceptional period and early stages of gestation can alter embryogenesis as preimplantation embryos are highly sensitive to elevated temperatures. In fact, many embryos do not survive heat-stress exposure, especially in the first week postfertilization (Sakatani et al., 2008). Further, exposure of Holstein heifers to heat stress during the first 7 d after estrus increased the proportion of abnormally and slowly developing embryos (Putney et al., 1989). This was more recently confirmed in vitro as exposure of culture of bovine zygotes to 40–40.5 °C reduced the percentage of zygotes that reached the blastocyst stage (Ortega et al., 2016). Abnormal development in bovine preimplantation embryos under hyperthermia is multifactorial. Maternal heat stress was reported to induce cytoplasmic changes such as reductions in mitochondrial membrane potential, a marker for developmental control in oocytes and preimplantation embryos, and in calcium ions levels, which has been associated with the impairment of cellular functions (Kamano et al., 2014). In addition, heat stress is associated with nuclear changes such as DNA fragmentation, a characteristic of apoptosis (Paula-Lopes and Hansen, 2002a). Part of the deleterious effects of elevated temperature on preimplantation embryos is also mediated by the increased production of reactive oxygen species, leading to a variety of cellular damages (de Barros and Paula-Lopes, 2018). Moreover, it is also possible that heat stress exposure at the zygote stage affects paternally imprinted genes, because paternal genome is the first one to be actively demethylated after fertilization (Oswald et al., 2000). Such susceptibility of the paternally imprinted genome to heat stress at this early stage could have immediate effects on embryo development, and errors in DNA methylation could be inherited by subsequent generations (de Barros and Paula-Lopes, 2018). However, this hypothesis warrants further investigation in dairy cows.

Embryos become more resistant to temperature as they advance in development. This thermotolerance was suggested to be related to the higher cell number as compared with the preimplantation embryo. It is also possible that the embryo acquires biochemical mechanisms of thermotolerance such as an increased protection by heat shock proteins as development progresses (Sakatani et al., 2013). Moreover, periconceptional heat stress could act as a means of natural selection causing only the best embryos to survive, thereby improving their thermotolerance (Rhoads, 2020). However, this hypothesis requires further research.

## Impacts of Maternal Heat Stress on Placental and Fetal Development

Maternal heat stress exposure during late gestation also impacts the developing fetus. Fetal temperature is maternally dependent until birth. Thus, alterations in maternal core temperature can impact fetal temperature. Maternal heat is transferred to the fetus via the placenta and the uterus. Conversely, heat generated by fetal metabolism is dissipated by the amniotic fluid to the uterine wall or via umbilical cord and placenta to maternal blood. The majority of heat is transferred through the placenta via the fetal–placental circulation, with only 10 to 20% dissipated via amniotic fluid (Kasiteropolou et al., 2020). Thus, prenatal fetal thermoregulation is facilitated by the maternal–fetal temperature gradient, fetal–maternal blood flow, and placental function and morphology.

During heat stress, maternal blood flow is diverted from the gravid uterus to the periphery in an effort to maximize maternal radiant heat loss thereby limiting the fetal temperature increment (Reynolds, 1990). Heat stress also decreases maternal dry matter intake in lactating pregnant cows, and in late-gestation dry cows, but to a lesser extent (Ouellet et al., 2020). Nutrition during pregnancy is a key determinant of placental growth. In addition, heat stress reduces blood concentrations of circulating placental hormones such as estrone sulfate, placental lactogen, and pregnancy-specific protein B reflecting an impairment of placental function, and development (Collier et al., 1982; Bell et al., 1989; Thompson et al., 2013). As the placenta is the organ for communication between mother and fetus, placental malfunctions invariably affect embryonic development and phenotypes in later life. Recent work revealed that a total of 169 genes were differentially expressed in placentae of pigs exposed to cyclic heat stress from day 40 to day 60 of gestation compared to placentae of pigs exposed to thermoneutral conditions (Zhao et al., 2021). Several of these genes were revealed to be involved in transport activity, glycoprotein biosynthetic processes, carbohydrate metabolic processes, solute carrier-mediated transmembrane transport, and glycosaminoglycan biosynthesis, which modulates placental stroma synthesis. Therefore, these authors identified altered placental nutrient transport capacity and metabolism as a possible mechanism for heat stress-induced placental inefficiency. Moreover, compensatory placental adaptations were reported in ewes exposed to hyperthermia during early pregnancy as the ratio of fetal to placental weight 15 to 20 d after the heat insult was approximately 40% higher compared to control ewes even though fetal weight was not different from that of control ewes. Increased expression of insulin-like growth factors (IGF-1), placental growth factor, and vascular endothelial growth factor in the placenta at day 55 of gestation suggests mechanisms for initial compensatory mechanisms (De Vrijer et al., 2006). Heat stress was also reported to reduce placental weight which is related to a decrease in tissue size rather than the number of placentomes (Early et al., 1991). Compared with those from sheep under thermoneutrality, the placenta from the hyperthermic animals had decreased total DNA, RNA, and protein content but concentrations were similar, which indicates that the reduced placenta mass is due to smaller cell number rather than cell size (Early et al., 1991). However, this remains to be demonstrated in dairy cows. Although nutrition is a key determinant of placental growth most studies suggest the negative effects of heat stress on placental and fetal growth are independent of nutrient intake (Bell et al., 1989; Alexander and Williams, 1971). To the best of our knowledge, there is currently a lack of investigations tracking the effects of periconceptional heat stress on placental function and morphology. Such investigations are important as the development of cotyledons, which transmit fetal blood and allow exchange of oxygen and nutrients with the maternal blood, starts early on in gestation (Van Eetvelde et al., 2016).

Novel work conducted in dairy cows indicated that late-gestation maternal heat stress can impact placental gross morphology with the potential to affect placental function. Late-gestation cows exposed to heat stress were reported to have an increased number of cotyledons, cotyledonary weights, and cotyledonary surface area and volume (Potadle et al., 2019). This could potentially indicate that the placenta responds to hyperthermia and nutrient restriction by a compensatory expansion of the cotyledonary surface. Interestingly, the increased cotyledonary surface associated with maternal heat-stress in dairy cattle does not seem to equate to higher nutrient and oxygen delivery to the fetus as IUHT calves are on average lighter at birth and have lower hematocrit relative to calves born to cooled dams (Monteiro et al., 2016a). However, conflicting results exist in the literature regarding the effects of late-gestation maternal heat stress on placental morphology in cattle. Recent work conducted at the University of Florida has demonstrated that placentae collected from late gestation heat-stressed dairy cows had lower cotyledons number and tended to have lower cotyledons surface relative to cows that were cooled during the last 2 mo of gestation (Casarotto et al., 2021). However, only a small subset of placentas was evaluated in the analysis. Placenta-related studies are challenging in the bovine, given that placentae are expelled within 2 to 12 h after parturition. The bovine placenta contains high levels of RNases (Burton et al., 2014). Hence, the length of the interval between separation from uterine wall, delivery, and tissue collection is a critical parameter to be considered when estimating gene expression (Burton et al., 2014).

Taken together, diverted blood flow, reduced nutrient intake, and alterations in placental morphology and function can create a nutrient-restricted hyperthermic intrauterine environment that limits fetal growth and eventually results in permanent adaptations (Ji et al., 2017). Moreover, maternal nutrition can have long-term metabolic consequences without necessarily affecting intrauterine growth (Bach et al., 2012).

## Impacts of Maternal Heat Stress on Birthweight and Growth

**Figure 2.**
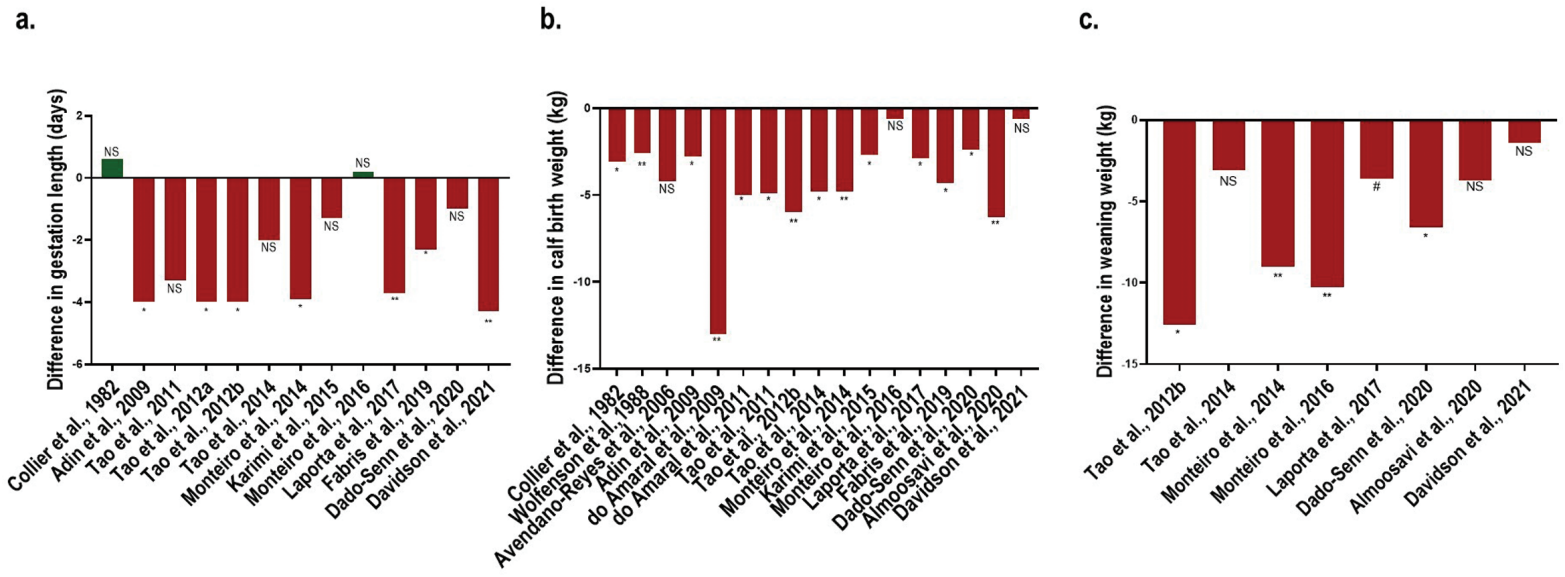
Summary of differences in (a) gestation length (days), (b) calf birth weight (kg), and (c) weaning weight (kg) between calves born to heat-stressed dams during the last stage of gestation and calves born to cooled dams during the last stage of gestation. NS indicates no significant difference between the two groups, ** indicates *P* < 0.01, and * indicates *P* < 0.05.

Birthweight of calves can act as a proxy of intrauterine growth. As far as we know, no controlled studies investigated the direct effects of periconceptional heat stress on calves’ birthweight and growth. However, a series of studies conducted in Holstein cows (Table 1) have demonstrated that late-gestation IUHT calves are born lighter relative to calves born to cooled dams (in utero cooled: **IUCL**) with a mean difference (±SD) of 4.2 ± 2.7 kg (Figure 2b). In the majority of studies, pregnant dams were reared in tropical or arid climates thus exposed to severe heat stress conditions. In order to discriminate between the direct effects of hyperthermia and indirect effects of reduced maternal feed intake on intrauterine growth, pair-feeding trials are required. Amongst all of the studies included in Figure 2b, only the one conducted by Almoosavi et al. (2020) followed a pair-fed experimental design. These authors reported that late-gestation IUHT calves were lighter at birth compared with calves born to cooled pair-fed dams indicating that intrauterine growth restriction under IUHT is independent of maternal feed intake.

**Table 1.** Main characteristics of studies included in the calculations to assess differences in gestation length, calf birthweight, and growth between late-gestation in utero heat-stressed Holstein calves and in utero cooled Holstein calves

References	Breed	Heat stress exposure days before calving	Location	Dam parity	Pair feeding
Collier et al. (1982)	Holstein	60	Bermuda	Multiparous	No
Wolfenson et al. (1988)	Holstein	60	Israel	Multiparous	No
Avendano-Reyes et al. (2006)	Holstein	60	Mexico	Multiparous	No
Adin et al. (2009)	Holstein	56	Israel	Multiparous	No
do Amaral et al. (2009)	Holstein	46	FL, USA	Multiparous	No
do Amaral et al. (2011)	Holstein	46	FL, USA	Multiparous	No
Tao et al. (2011)	Holstein	46	FL, USA	Multiparous	No
Tao et al. (2012a)	Holstein	46	FL, USA	Multiparous	No
Tao et al. (2012b)	Holstein	45	FL, USA	Multiparous	No
Tao et al. (2014)	Holstein	45	FL, USA	Multiparous	No
Monteiro et al. (2014)	Holstein	46	FL, USA	Multiparous	No
Karimi et al. (2015)	Holstein	21	Iran	Multiparous	No
Monteiro et al. (2016)	Holstein	45	FL, USA	Multiparous	No
Guo et al. (2016)	Holstein	45	FL, USA	Multiparous	No
Laporta et al. (2017)	Holstein	46	FL, USA	Multiparous	No
Fabris et al. (2019)	Holstein	45	FL, USA	Multiparous	No
Dado-Senn et al. (2020)	Holstein	44	FL, USA	Multiparous	No
Almoosavi et al. (2020)	Holstein	45	Iran	Multiparous	Yes
Davidson et al. (2021)	Holstein	60	FL, USA	Nulliparous	No

A reduced gestation length can also contribute to the observed intrauterine growth restriction and reduced birthweights under late-gestation IUHT. Dams exposed to heat stress during the last 46 d of gestation calved on average (±SD) 2.5 ± 1.6 d earlier compared to dams provided with active cooling during the last weeks of gestation (Figure 2a). However, two studies found no difference in gestation length of late-gestation dams exposed to heat stress or provided with cooling whereas they reported a difference in calf birth weight (Collier et al., 1982; Karimi et al., 2015). This indicates that intrauterine growth may also be independent of a shorter gestation length.

Skibiel et al. (2018a) examined morphology and DNA methylation changes of the liver in late-gestation IUHT and IUCL bull calves at birth. The authors reported that the liver of late-gestation IUHT calves contained more cells relative to IUCL calves, and exhibits 239 differentially methylated genes. These findings point toward an epigenetic component to postnatal phenotypic differences observed at birth in late-gestation IUHT and IUCL calves. However, the authors could not assess the contribution of other epigenetic mechanisms besides DNA methylation such as histone modifications (i.e., acetylation and ubiquitination), chromatin remodeling, or microRNAs.

Late-gestation maternal heat stress exerts carry-over effects on progeny growth. Consistently, many studies reported differences in weaning weight between IUHT and IUCL calves with an average (±SD) difference of 6.3 ± 3.7 kg (Figure 2c). Dado-Senn et al. (2020b) assessed the impact of both prenatal and postnatal heat stress exposure on calf productivity, including preweaning average daily gain (**ADG**). These authors reported that ADG was driven entirely by prenatal heat stress. Furthermore, a retrospective study by Monteiro et al. (2016b) reported that the impact of IUHT on heifer body weight persisted through 1 yr of age, whereby IUHT heifers weighed less every month up to 12 mo of age relative to IUCL heifers. Of interest, that difference was gone by 2 yr of age when the heifers calved for the first time, suggesting some compensatory gain.

## Effects of Maternal Heat Stress on Thermotolerance

Maternal heat stress during critical embryonic and fetal development stages could potentially result in the selection of the fittest embryos, thereby improving thermotolerance during postnatal life, and offering an advantage to the heat stress-conceived cows during subsequent periods of heat stress (Rhoads, 2020). While we could not find any studies about the effects of maternal periconceptional heat stress on thermotolerance in dairy cows, Ahmed et al. (2017) tested the hypothesis that late-gestation maternal heat stress improves heat tolerance at maturity in dairy cows. Relative to control animals, late-gestation IUHT animals had limited response to acute heat stress at maturity reflecting a higher thermotolerance. This thermotolerance is mainly accomplished by an increased blood flow to the skin in IUHT animals, which supports body temperature regulation without activating the sweating response. Moreover, IUHT animals potentially have a hair coat that facilitates heat exchange to the environment. On the contrary, Dado-Senn et al. (2020b) reported that late-gestation IUHT dairy calves had greater rectal temperature and respiration rate compared to IUCL animals. These recent results are consistent with work conducted in pigs whereby pigs exposed to IUHT had a preprogrammed increase in postnatal core body temperature set-point that remained elevated regardless of ambient temperature exposure (Johnson et al., 2015). A greater core body temperature would likely compromise thermotolerance under postnatal heat stress as heat loss relies partly on maintaining a thermal gradient between the animal and the environment. The increase of core body temperature in IUHT pigs under postnatal heat stress is potentially associated with an increase in metabolic heat production and an increase in backfat, which can trap metabolic heat (Johnson et al., 2015). Differences in thermotolerance between late-gestation IUHT calves before weaning and IUHT mature lactating cows can be attributed to difference in physiological state and to the match/mismatch theory associated with developmental programming (Rhoads, 2020). This theory implies that matched prenatal/postnatal environments confer advantageous adaptations whereas mismatched prenatal/postnatal are generally detrimental to the offspring. Additional investigation is required to understand the basis for periconceptional and late-gestation maternal heat stress on thermotolerance in IUHT cows.

## Effects of Maternal Heat Stress on Metabolism and Immune Response

To the best of our knowledge, no studies track the effects of periconceptional heat stress on calf metabolism and immune response. In contrast, several studies report that late-gestation IUHT calves have lower plasma concentrations of insulin, prolactin, and insulin-like growth factor-I but no change in glucose, nonesterified fatty acids, or b-hydroxybutyrate plasma concentrations within 2 h after birth. In addition, after colostrum consumption, late-gestation IUHT calves have greater circulating insulin in the first and second weeks of life relative to IUCL calves (Monteiro et al., 2016b; Almoosavi et al., 2020). However, this difference in circulating insulin does not persist as the calf develops postnatally (Monteiro et al., 2016b). Collectively, investigations conducted on the effects of late-gestation maternal heat stress on calf metabolism indicate that suboptimal intrauterine conditions can program the metabolism of the fetus as IUHT induces insulin resistance in peripheral tissues, but improves the progeny’s postnatal insulin-independent glucose absorption and basal glucose uptake.

It is also widely reported that late-gestation maternal heat stress influences immune function of the offspring despite similar maternal colostrum IgG concentrations. Indeed, IUHT calves have lower serum IgG concentration at and after 24 h of birth and lower apparent efficiency of IgG absorption relative to IUCL calves, suggesting that IUHT impairs passive immunity (Monteiro et al., 2014; Laporta et al., 2017). This was recently confirmed in a study conducted in calves born to late gestation heat-stressed nulliparous heifers whereby apparent efficiency of absorption of IgG tended to be lower in late-gestation IUHT calves compared with IUCL calves whereas serum IgG concentrations from birth to day 56 were significantly lower (Davidson et al., 2021). Moreover, Almoosavi et al. (2020) demonstrated that the lower serum concentration of IgG and apparent efficiency of IgG absorption in IUHT calves is independent of reduced maternal dry matter intake. Potential reasons behind the impairment of passive transfer in late-gestation IUHT calves are reduced postnatal absorption in the small intestine, as a result of impaired intestinal development and decreased surface area for absorption due to intrauterine growth retardation. Ahmed et al. (2021) suggest that impaired passive immune transfer in IUHT calves is a consequence of reduced enterocyte turnover in the small intestine over the first days of life. Further, it cannot be excluded that the decrease in gestation length associated with late gestation IUHT may prevent intestinal enterocytes in reaching their full endocytotic potential.

## Effects of Maternal Heat Stress on Production Outcomes

**Figure 3.**
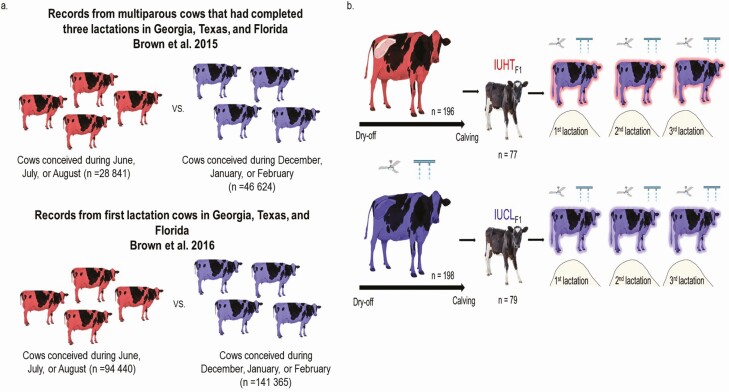
Schematic representation of the experimental design followed by (a) Brown et al. (2015, 2016) and (b) Laporta et al. (2020) to track the production outcomes of in utero heat-stressed cows (IUHT_F1_) during a. the periconceptional period and b. late-gestation. Brown and collaborators compared the production outcomes of cows that conceived within the months of June, July, and August (red; heat stress) to those that conceived within the months of December, January, and February (blue; thermoneutral). Laporta and collaborators conducted a retrospective analysis across a 10-yr period from studies comparing the production outcomes of late-gestation IUTH cows (red) to late-gestation IUCL cows (blue). Animals of both groups were managed in a similar manner during postnatal life.

A few research reports have tracked the production outcomes of dairy cows whose dams were exposed to heat stress around the time of conception or during late gestation. Brown et al. (2015, 2016) compared the milk production of cows that conceived within the months of June, July, and August (heat stress) to those that conceived within the months of December, January, and February (thermoneutral) in Georgia, Florida, and Texas in the United States (Figure 3a). For their part, Laporta et al. (2020) conducted a retrospective analysis across a 10-yr period from studies using the same experimental design of late-gestation maternal heat stress to compare reproductive and productive performance and survival of daughters whose dams were actively cooled or heat-stressed during the last 46 d of gestation (Figure 3b). The authors reported that reproductive performance was similar between late-gestation IUHT and IUCL cows. The lack of effect of late-gestation IUHT on reproductive performance may be related to the timing of maternal heat stress exposure in late gestation. Primordial germ cells in the bovine fetus differentiate into oogonia during the first trimester of gestation and divide mitotically well into the second trimester of gestation (Bras, 2008). Thus, the first and second trimesters are determinant for the future reproductive performance of the female. In contrast, Chavez et al. (2020) observed a reduction in reproductive competence in heifers that were IUHT in the final trimester of pregnancy relative to those experiencing cool conditions. Possible explanation for this decrease includes a lower ovarian reserve as reflected by lower anti-Müllerian hormone reported in IUHT calves (Akbarinejad et al., 2017). Unfortunately, reproductive performance was not evaluated in the studies conducted by Brown et al. (2015, 2016). Yet, more research imposing maternal heat stress during lactation is necessary to better understand the effects of maternal heat stress on the future reproductive performance of the progeny.

Brown et al. (2015, 2016) reported that thermoneutral-conceived cows outperformed their heat stress-conceived counterparts. Thermoneutral-conceived cows from the primiparous analyses produced 172 ± 43 to 423 ± 39 kg more milk compared to the heat stress-conceived cows while multiparous thermoneutral-conceived cows produced between 82 ± 42 and 399 ± 61 kg more milk than their heat stress-conceived counterparts. The discrepancies in the extent of the production difference between the thermoneutral and heat-stress groups of 2015, and 2016 studies are most likely due to the more restrictive inclusion criteria in the 2016 study as only primiparous cows were included in the analysis.

Laporta et al. (2020) reported that late-gestation IUHT can also impact the lactational performance of the resulting offspring up to three lactations. When considering a lactation of 305 d, milk production of late-gestation IUHT was reduced on average by 671, 702, 1,983 kg/lactation in their first, second, and third lactations, respectively, compared with IUCL animals. More IUHT daughters were also culled before first calving, and the productive life and lifespan were reduced relative to daughters born from cooled dams (4.9 and 11.7 mo, respectively). Further, the granddaughters (F2) born to these IUHT daughters also produced less milk in their first lactation (on average 397 kg/lactation) relative to F2 born to IUCL daughters. Besides the effects on milk production, late-gestation IUHT reduced the length of productive life and lifespan of both the daughters and granddaughters of dams exposed to hyperthermia in late gestation.

The effects of maternal heat stress on milk production and survival of the resulting offspring are multifaceted and are potentially the culmination of the aforementioned inefficient phenotypes. Furthermore, although not confirmed experimentally in their studies, Brown et al. (2015, 2016) suggested that the milk production decrease observed in cows born to periconceptional heat-stressed dams is a direct result of the match/mismatch phenomenon between prenatal and postnatal environment that governs the outcomes of developmental programming. In addition, Skibiel et al. (2018b) reported that, although having a similar number of alveoli, alveolar area was reduced in late-gestation IUHT heifers in their first lactation, compared with IUCL. Given the positive correlation between alveoli area and the number of mammary epithelial cells they contain, the lower alveoli area indicates lower milk storage and milk synthesis capacity and may explain, in part, the reduced milk production observed in IUHT cows. Further, Skibiel et al. (2018a) found that late-gestation IUHT alters the methylation pattern of mammary tissue collected during the first lactation of IUHT daughters, reinforcing the idea that IUHT exerts long-lasting epigenetic changes in the mammary gland.

## Future Perspective

Significant progress has been made in identifying maternal heat stress consequences on the resulting progeny in dairy cows and in understanding its epigenetics implications. Most of the studies discussed in this review were carried out in Holstein cows considering maternal heat stress in the last stage of gestation. There are opportunities for research discoveries from new emerging work targeting the first stage of gestation as the initial development and establishment of the organs occurs in the first 90 to 180 d of gestation. Moreover, future studies examining the effects of maternal heat stress should also measure the epigenetic alterations besides methylation and other molecular changes that could be triggered by maternal heat stress. This is critical to understand the physiological mechanisms behind the production impairment associated with maternal heat stress on the resulting progeny. Furthermore, the consequences of maternal heat stress on the dam itself and on the progeny described in this review highlighted the relevance of the development of heat-tolerant dairy cows to reduce the impact of heat stress and ensure the durability of the dairy sector worldwide.

## Conclusions

It is now clear that the intrauterine environment plays a crucial role in determining phenotypes of dairy cows in later life. Maternal exposure to environmental heat stress during embryonic and fetal development leads to changes in intrauterine growth, epigenome, thermotolerance, metabolism, organ development, and immune response in the resulting progeny. In turn, the combinations of these altered phenotypes translate into lower milk production and survival, which may be recapitulated in further generations. New insights point toward an epigenetic component to the reported inefficient postnatal phenotypes. From a practical point of view, these findings have important implications to adapt dairy farms in the context of climate change to ultimately maintain the durability of the dairy sector.

## About the Authors

**Veronique Ouellet**, PhD, is an assistant professor at Laval University in the Department of Animal Sciences where she teaches Dairy Production. Her recent research interests have focused on improving dairy herd resiliency to heat in a continental climate. Furthermore, she is also interested in developing technologies and strategies to help dairy producers to adapt their dairy farms to climate change.

**Alexandra Boucher** received her BSc in agronomy from Laval University. She has recently started her master’s degree in animal sciences. She is primarily interested in the effects of environmental and social stressors on dairy cows and their effects on productivity and longevity of the herd.

**Geoffrey E. Dahl** is the Harriet B. Weeks Professor in the Department of Animal Sciences at the University of Florida, Gainesville. He conducts applied and basic research with direct impact on dairy production and management. Specific research interests include effects of photoperiod manipulation on production and health, the impact of frequent milking in early lactation on milk production, and heat stress abatement during the dry period on cow productivity and health. Recent efforts have focused on the effects of in utero heat stress on the performance and health of the calf.

**Jimena Laporta** received her PhD in Dairy Science from UW–Madison and was a faculty member in the Department of Animal Sciences at the University of Florida for 5 yr. She joined the Department of Animal and Dairy Sciences at UW–Madison in 2020 as an Assistant Professor in lactation physiology. Her research incorporates state-of-the-art molecular techniques to investigate how autocrine and environmental factors affect mammary gland function and how maternal influences during gestation might program the developing fetus.
